# Assessment of More Sustainable Packaging Solutions for Sliced Cooked Ham and Mortadella: A Study on Physicochemical Properties and Microbial Quality

**DOI:** 10.1111/1750-3841.70574

**Published:** 2025-09-28

**Authors:** Giulia Leni, Cansu Ekin Bonacina, Laura Principato, Roberta Galli, Carlo Negrini, Giuliano Dallolio, Andrea Bassani, Giorgia Spigno

**Affiliations:** ^1^ Department for Sustainable Food Process (DiSTAS) Università Cattolica del Sacro Cuore Piacenza Italy; ^2^ Department of Food Engineering, Faculty of Engineering Ankara University Ankara Turkey; ^3^ Gianni Negrini srl Renazzo Italy

## Abstract

The growing demand for alternative food packaging materials has gained significant attention, driven by both European and global policies aimed at promoting a more sustainable food system. In the present work, more sustainable packaging materials were compared to a conventional system (tray in PET/EVOH/PE with lid in PET/EVOH/PE) to preserve pre‐cut cooked ham and mortadella packaged under a modified atmosphere during storage. The alternative systems included a tray in PET/R‐PET/PET with a BOPET lid (System 2) or a lid in PET‐AlOx/PET (System 3) and a tray in BIOPET/R‐PET/PET with a BIOPET lid (System 4). The results revealed that the packaging systems, storage time (performed at 4°C), and light/dark conditions significantly affected the physicochemical properties and microbiological stability of both processed meat products. In particular, cooked ham stored in System 1 exhibited the best color retention, with a* and b* color coordinates slightly degrading over time. Mortadella samples, on the contrary, appeared less affected by discoloration phenomena, with System 2 under light conditions performing the worst in preserving the product. *a*_w_ and pH decreased in cooked ham and mortadella packaged in all systems throughout the storage period, with light exposure accelerating these changes. Finally, microbiological analysis demonstrated that alternative System 2 performed similarly to conventional System 1 in controlling microbial growth during product storage. The results highlight the importance of thoroughly exploring the effects of more sustainable packaging materials to maintain product quality, particularly in terms of color, *a*_w_, and pH.

**Abbreviations**
*a*_w_
: water activity
LAB: lactic acid bacteria
MAP: modified atmosphere packaging
MDA: malondialdehyde
OTR: oxygen transfer rate
pack: packaging
SE: standard error
TBARS: thiobarbituric acid reactive substances
TMC: total microbial count
WVTR: water vapor transfer rate

## 1 Introduction

In recent years, the progress in food packaging has been growing rapidly to respond to a global need for more environmentally friendly materials (Mahmud et al. 2024). Traditional petroleum‐based plastics, with their favorable barrier properties and durability, are being reconsidered in favor of more sustainable alternatives (Yin and Woo 2024). In this context, bioplastics and recyclable materials aim to reduce the footprint of the food packaging sector (Shlush and Davidovich‐Pinhas 2022). In addition, mono‐material packaging systems, composed of a single polymer type, are generally considered more sustainable due to their improved recyclability and lower environmental impact throughout the lifecycle (Geueke et al. 2018). In contrast, conventional multilayer packaging, while offering excellent barrier properties, often hinders recycling processes and increases plastic waste (Tamizhdurai et al. 2024). The transition towards a more sustainable food packaging system is also encouraged by consumers, who are becoming increasingly concerned about packaging sustainability and are willing to pay more for packaging they perceive as environmentally friendly (Herrmann et al. 2022). However, the main challenge remains the development of sustainable packaging solutions that are able to ensure food quality, safety, and shelf life, at least on par with conventional materials (Bonacina et al. 2025).

Within the meat sector, various strategies have been employed to preserve food quality, with vacuum or modified atmosphere packaging (MAP) and high‐barrier plastic materials used for product preservation (Garcia et al. 2024). In this regard, the integration of more sustainable alternatives remains challenging, since the barrier properties of alternative materials are often inferior when compared to conventional plastics (Cenci‐Goga et al. 2020). In particular, effective gas barrier properties are essential for controlling oxidation, moisture, and gas exchange, and, as a cascade effect, microbial growth. In addition, factors such as light exposure and storage conditions also contribute to influencing meat quality and shelf life (Nethra et al. 2023). This is not of secondary importance, since meats are highly perishable products, being dynamic systems that undergo deterioration and chemical changes (Nethra et al. 2023). In particular, discoloration and microbial growth are considered the most important factors responsible for meat deterioration (Iulietto et al. 2015). Discoloration of meat occurs due to lipid oxidation and can be prevented by introducing additives (e.g., salt and nitrite) and using MAP or vacuum packaging (Garcia et al. 2024). Furthermore, microbial growth influences meat safety and, with their metabolism, the overall quality, with a series of chemical reactions causing oxidation, off flavors, texture, and color changes (Garcia et al. 2024).

Based on these premises, the aim of this study was to evaluate the performance of alternative, more sustainable plastic materials, compared to a conventional solution, in preserving the quality of pre‐sliced processed meat products. In particular, pre‐cut cooked ham and mortadella were packaged in MAP, and their quality parameters were monitored during refrigerated storage under light or dark conditions. Thus, this research aims to assess whether more sustainable packaging systems could effectively preserve the quality parameters of pre‐sliced processed meat products.

## 2 Materials and Methods

### 2.1 Sample

The samples were represented by cooked ham and mortadella, manufactured and packaged by an Italian factory located in the Emilia‐Romagna region. The cooked ham was produced from 100 kg of heavy Italian pork hams and 25 kg of brine (20% w/w; containing water, salt, sodium nitrite, E301 antioxidant, and natural flavors), using a multi‐needle injector. The product was then massaged in a tumbler and placed in molds for steam cooking until a core temperature of 69°C was reached. Subsequently, it was cooled, vacuum‐packed, and pasteurized at 105°C for 20 min. According to the producer's label, cooked ham contained 67% moisture, 11% fat (of which 4% was saturated), 18% protein, 1% carbohydrates, and 3% ash. Mortadella, on the other hand, was produced from Italian pork meat, pork tripe, salt, sucrose, natural flavors, spices, antioxidant E301, and sodium nitrite, in accordance with the “Mortadella Bologna PGI” disciplinary (MASAF 2025). Briefly, the process includes grinding using a mincer equipped with a final plate having holes no larger than 0.9 mm, followed by mixing. The mixture was then stuffed in a casing permeable to water vapor, cooked in a dry air oven until reaching a core temperature of 70°C, and finally subjected to rapid cooling. According to the producer's label, mortadella contained 57% moisture, 24% fat (of which 8.3% was saturated), 16% protein, and 3% ash. For the experiments, cooked ham and mortadella were sliced at a thickness of 2 mm and packaged in rigid trays under MAP, with 20% CO_2_ and 80% N_2_, containing 100 ± 5 g of product.

Four different packaging solutions were selected based on the options provided by the supplier, considering the monomaterial/recycled material characteristics and the barrier properties. Three of these solutions represented a potential alternative to the conventional system. The packaging solutions were presented in Table 1, with information regarding their barrier properties (data provided by the supplier or obtained through external analysis).

**TABLE 1:** Packaging systems used for the storage of both sliced cooked ham and mortadella samples, including information on the tray's oxygen transfer rate (OTR), lid OTR and water vapor transfer rate (WVTR).

Packaging systems	Tray composition	Tray OTR cm^3^/pkg/24h	Lid composition	Lid WVTR g/pkg/24h	LidOTR cm^3^/pkg/24h
1	PET/EVOH/PE	0.1^a^	PET/EVOH/PE	10^b^	2.0^a^
2	PET barrier/R‐PET/PET	<0.05^c^	BOPET	1.7^d^	1.3^e^
3	PET barrier/R‐PET/PET	<0.05^c^	PET‐AlOx/PET	1.9^f^	2.8^e^
4	bioPET/R‐PET/PET	0.1^c^	BOPET	1.7^d^	1.3^e^

^a^ Measured with ASTM D3985.

^b^ Measured with ASTM F124.

^c^ Measured with ASTM F 1307–20.

^d^ Measured with ASTM E96/E96M‐16.

^e^ Measured with ASTM D3985‐17.

^f^ Measured with ASTM E96/E96M‐17.

Abbreviation: pkg, packaging.

**FIGURE 1:**
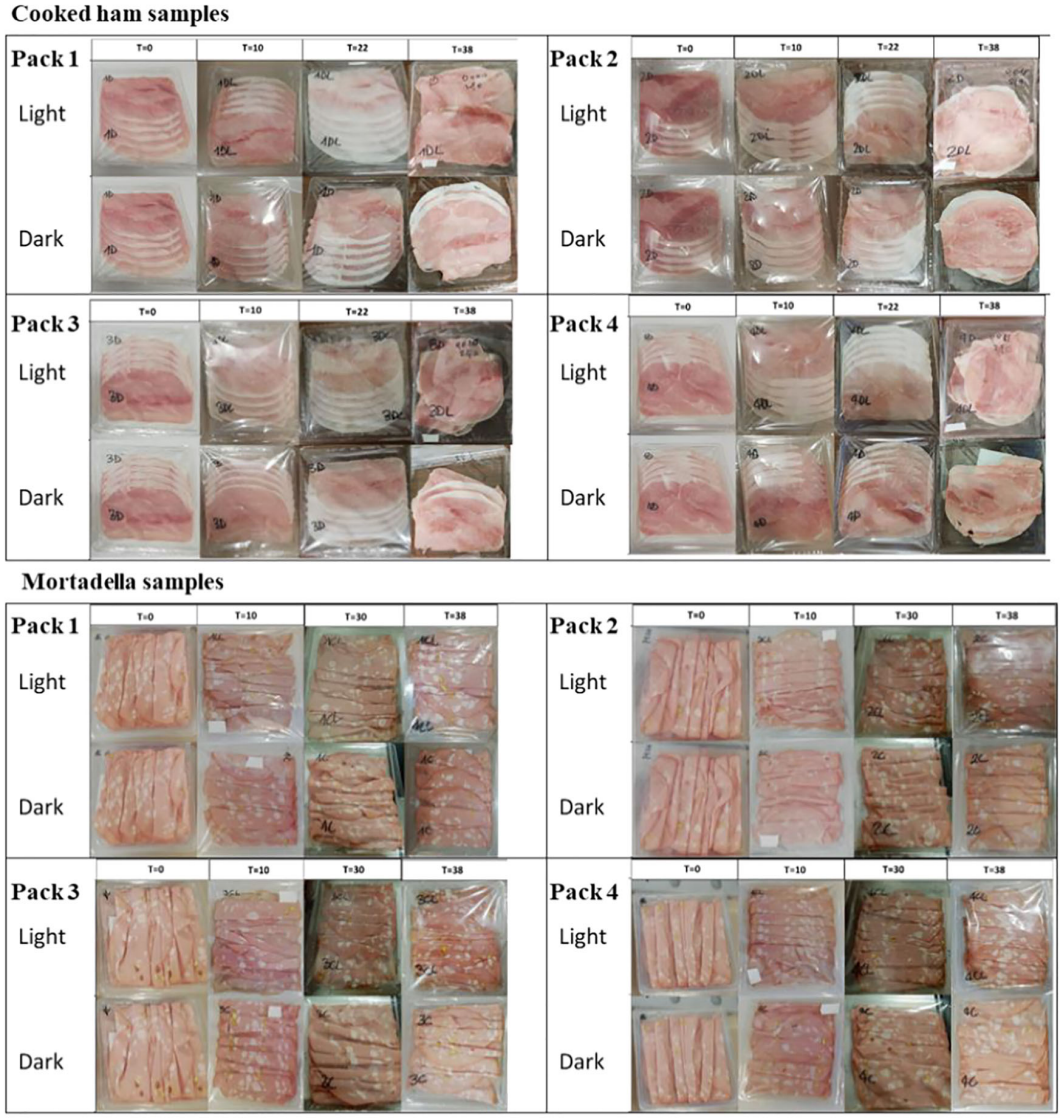
Images of cooked ham and mortadella samples during their storage in the different packaging systems, under both light and dark conditions.

Packaging solution 1 was the conventional system currently used by the food company, while solutions 2, 3, and 4 were considered alternative packaging systems. Samples were stored at 4°C, and their evolution was monitored at six different time points during the 38‐day shelf life: Day 0, 3, 10, 22, 30, and 38 (Figure 1).

Half of the samples (144 in total) were stored under dark conditions through the use of an HPP260eco climatic chamber from Memmert (Büchenbach, Germany), with a relative humidity of around 87%, simulating the domestic refrigerator conditions. The other half (144 samples) were stored under the same conditions but irradiated with artificial light, as proposed by Haile et al. (2013). For irradiation, linear fluorescent lamps (1000 lx emission) were used to simulate actual display conditions of the product in retail sales. Samples were rotated during the monitoring period in order to uniformly distribute the light level exposure conditions.

### 2.2 Analysis

#### 2.2.1 CO_2_ Headspace Analysis

The samples were subjected to the measurement of the percentage of carbon dioxide (CO_2_) through the Dansensor CheckMate 3 gas meter (Ametek, Ripalta Cremasca, CR). The analysis was carried out by piercing the packaging with a special needle that allows the detection of the percentage of gases within 2–3 seconds.

#### 2.2.2 Color Analysis

CIELAB color analysis was performed with the HunterLab D25 NC colorimeter (Hunter Associates Laboratory, Reston, Virginia, USA), equipped with a sensor with a diameter of 25.4 mm after 30 min blooming time. The coordinates are detected by 5 flashes per second. The calibration of the HunterLab D25 NC, carried out at each switch‐on, was carried out by means of a white ceramic tile that has the following coordinates: *Y* = 86.75, *X* = 81.70, *Z* = 92.71. For this analysis, the slice on the top, two central slices, and the bottom slice were used in order to obtain average values.

#### 2.2.3 Water Activity, pH, and Lipid Oxidation Measurement (TBARS Assay)

For the analyses, all slices within the same tray were ground together with a laboratory grinder (Moulinex Essential DJ5201, Groupe Seb, Écully, France) and considered representative of each tray.

Water activity (*a*_w_) of samples was measured by the instrument HygroPalm23‐A (Rotronic Italia, Rho, MI) with Rotronic Hw4 recognition software. Each container was filled to about two‐thirds of its volume with ground sample.

The pH measurements were done by using the pH‐meter Sension+ PH3 (Hach Instruments, Lainate, MI). For this analysis, a ground sample was placed in paper cups with 50 mL of 7.5% w/v KCl in water. The content was mixed and homogenized on the vortex Velp Magnetica (Usmate Velate, MB) for about 2 min, at a speed of 300–400 rpm. Finally, the mixture was rested for 5 min before pH measurement in order to stabilize the result. The calibration of the instrument was performed with buffer solutions at pH 4 and 7.

Lipid oxidation was evaluated using the thiobarbituric acid reactive substances (TBARS) assay, which involves the quantification of aldehyde, expressed as malondialdehyde (MDA) equivalents per kg of product (mg eq MDA/kg). For the analysis, 10 g of ground samples were placed in a stomacher bag with 50 mL of 10% trichloroacetic acid (TCA). Samples were homogenized and subsequently filtered with Whatman paper filter no. 1. An equivalent volume of 0.02 M 2‐thiobarbituric acid was added, and the solution was kept in the dark and at room temperature for about 24 h. The measurement was carried out using the UV‐visible spectrophotometer at 530 nm (spectrophotometer V‐730, Jasco, UK), reading the samples against the blank solution of TCA: Thiobarbiturate (1:1). The absorbance was converted to MDA concentration by means of a calibration curve (*R* ^2^ = 0.9965) obtained with MDA (concentration range 0.05–0.5 µM).

### 2.3 Microbiological Analysis

Microbiological analyses were conducted exclusively on the set of samples stored in the dark, to simulate potential microbial growth that could occur in a domestic environment. These analyses were performed on ground samples, prepared as described in Section 2.2.3, at time points 0, 10, 22, and 30 days, with the final time point corresponding to the end of the product's declared shelf life. The microbial measurements included the total microbial count (TMC) at 30°C (ISO 4833:2013), along with psychrotrophs (ISO 17410:2019), lactic acid bacteria (LAB) (ISO 15214:1998), coagulase‐negative staphylococci (ISO 6888‐1:2021), Enterobacteriaceae (ISO 21528‐2:2017), sulfite‐reducing Clostridia (ISO 15213‐2:2023), *Bacillus cereus* (ISO 7932:2004), and yeast and molds (ISO 21527:2008).

### 2.4 Statistical Analysis

A total of 144 samples of packaging containing cooked ham and 144 packaging containing mortadella samples were obtained. In particular, three replicates for each combination of packaging type (four systems for both products), light condition (dark and light), and storage time points (six time points during the monitoring period). The results were reported as mean values ± standard errors. Statistical analyses were performed by SPSS Statistics 25 software (IBM, USA). The effect of the packaging system, storage time, and light/dark conditions, and their first‐ and second‐order interactions with the measured parameters were assessed by three‐way ANOVA. In the case of a significant difference, the means were discriminated by applying Tukey's post hoc test. Significant differences were compared at a level of *p* < 0.05.

## 3 Results and Discussion

The significance of the interaction and main effects of the packaging system, light/dark storage conditions, and storage time for all investigated parameters for both cooked ham and mortadella samples are listed in Table 2.

**TABLE 2:** ANOVA *p*‐values for main and interaction effects of packaging type, time of storage and light/dark conditions on the different parameters measured for both pre‐sliced cooked ham and mortadella samples.

	Parameters	Pack	Time	Light	Pack * Light	Pack * Time	Light * Time	Pack * Time * Light
**Cooked ham**	**CO_2_**	***	NS	***	NS	NS	NS	*
	L*	**	***	NS	*	NS	NS	NS
	a*	***	***	***	NS	*	NS	NS
	b*	NS	***	NS	NS	NS	NS	NS
	***a*_w_**	***	***	***	***	***	***	NS
	**pH**	***	***	***	***	***	***	***
	**TBARS**	***	***	***	***	***	***	***
**Mortadella**	**CO_2_**	***	***	***	***	*	*	***
	L*	NS	***	**	NS	NS	NS	NS
	a*	NS	***	***	*	NS	NS	NS
	b*	NS	***	**	NS	NS	NS	NS
	***a*_w_**	NS	***	***	NS	*	***	**
	**pH**	NS	***	NS	NS	NS	**	NS
	**TBARS**	**	***	NS	NS	NS	*	NS

Abbreviations: *a*_w_, water activity; pack, packaging; TBARS, thiobarbituric acid reactive substances.

^NS^ *p* > 0.05; *p < 0.05; **p < 0.01; ***p < 0.001.

### 3.1 CO_2_ Headspace Analysis

A significant interaction (*p *< 0.05) between packaging, light/dark conditions, and storage time was determined for CO_2_ in both pre‐sliced mortadella and cooked ham samples (Table 2). For cooked ham samples stored under dark conditions, a significant reduction in CO_2_ content (*p *< 0.05) during the storage period was observed, as reported in Table 3.

**TABLE 3:** Effect of packaging material and light/dark condition on CO_2_ levels, pH, thiobarbituric acid reactive substances (TBARS), and water activity (*a*_w_) over the storage period.

			Storage period (days)						
		Pack	0	3	10	22	30	38	SE
**Cooked ham CO_2_** **%**	**Dark**	**1**	23.77 ^cdefg^	23.13 ^abcdefg^	23.43 ^bcdefg^	23.00 ^abcdefg^	23.47 ^bcdefg^	24.13 ^defg^	0.14
		**2**	22.77 ^abcdefg^	21.43 ^abcd^	21.73 ^abcd^	21.37 ^abcd^	20.90 ^ab^	21.00 ^abc^	
		**3**	24.00 ^defg^	23.20 ^bcdefg^	21.87 ^abcde^	21.73 ^abcd^	22.10 ^abcdef^	22.00 ^abcde^	
		**4**	22.17 ^abcdef^	21.17 ^abc^	21.47 ^abcd^	20.40 ^a^	21.83 ^abcde^	21.13 ^abc^	
	**Light**	**1**	23.77 ^cdefg^	24.60 ^efg^	24.83 ^fg^	27.77 ^hi^	28.03 ^i^	29.57 ^i^	0.26
		**2**	22.77 ^abcdefg^	22.37 ^abcdefg^	22.63 ^abcdefg^	22.77 ^abcdefg^	22.80 ^abcdefg^	25.13 ^gh^	
		**3**	24.00 ^defg^	24.03 ^defg^	23.97 ^defg^	23.53 ^bcdefg^	23.03 ^abcdefg^	21.90 ^abcde^	
		**4**	22.17 ^abcdef^	22.93 ^abcdefg^	21.97 ^abcde^	21.70 ^abcd^	21.67 ^abcd^	22.77 ^abcdefg^	
**Mortadella CO_2_** **%**	**Dark**	**1**	24.57 ^ghij^	24.30 ^fghij^	24.23 ^efghij^	22.50 ^bcdefgh^	21.33 ^b^	18.53 ^a^	0.17
		**2**	24.93 ^ij^	23.43 ^bcdefghij^	23.40 ^bcdefghij^	23.43 ^bcdefghij^	22.30 ^bcdefg^	22.37 ^bcdefg^	
		**3**	24.57 ^ghij^	23.53 ^bcdefghij^	23.00 ^bcdefghij^	22.33 ^bcdefg^	21.57 ^bc^	21.83 ^bcd^	
		**4**	24.13 ^defghij^	24.77 ^hij^	23.03 ^bcdefghij^	23.10 ^bcdefghij^	22.73 ^bcdefghij^	22.33 ^bcdefg^	
	**Light**	**1**	24.57 ^ghij^	24.77 ^hij^	24.50 ^ghij^	24.20 ^defghij^	23.93 ^cdefghij^	23.70 ^bcdefghij^	0.15
		**2**	24.93 ^ij^	25.00 ^j^	23.57 ^bcdefghij^	23.60 ^bcdefghij^	22.90 ^bcdefghij^	22.07 ^bcdef^	
		**3**	24.57 ^ghij^	23.21 ^bcdefghij^	23.15 ^bcdefghij^	22.57 ^bcdefghi^	21.90 ^bcde^	21.47 ^b^	
		**4**	24.13 ^defghij^	23.50 ^bcdefghij^	23.23 ^bcdefghij^	23.07 ^bcdefghij^	22.87 ^bcdefghij^	22.23 ^bcdefg^	
**Cooked ham pH**	**Dark**	**1**	6.37 ^fgh^	6.37 ^fgh^	6.36 ^fgh^	6.14 ^defgh^	6.19 ^efgh^	6.13 ^defgh^	0.01
		**2**	6.44 ^gh^	6.44 ^fgh^	6.36 ^fgh^	6.25 ^efgh^	6.40 ^fgh^	6.17 ^defgh^	
		**3**	6.37 ^fgh^	6.34 ^efgh^	6.32 ^efgh^	6.27 ^efgh^	6.28 ^efgh^	6.17 ^defgh^	
		**4**	6.46 ^h^	6.43 ^fgh^	6.43 ^fgh^	6.35 ^efgh^	6.23 ^efgh^	6.18 ^efgh^	
	**Light**	**1**	6.37 ^fgh^	6.41 ^fgh^	6.34 ^efgh^	5.81 ^bcd^	5.48 ^ab^	5.34 ^a^	0.04
		**2**	6.44 ^gh^	6.42 ^fgh^	6.43 ^fgh^	6.11 ^defgh^	5.98 ^cde^	5.70 ^abc^	
		**3**	6.37 ^fgh^	6.31 ^efgh^	6.29 ^efgh^	6.07 ^cdef^	6.09 ^defgh^	6.09 ^defgh^	
		**4**	6.46 ^h^	6.35 ^fgh^	6.35 ^efgh^	6.30 ^efgh^	6.20 ^efgh^	6.08 ^defg^	
**Cooked ham TBARS** **mg eq MDA/kg**	**Dark**	**1**	0.06 ^a^	0.07 ^a^	0.05 ^a^	0.12 ^ab^	0.12 ^ab^	0.08 ^a^	0.01
		**2**	0.08 ^a^	0.06 ^a^	0.06 ^a^	0.11 ^ab^	0.08 ^a^	0.09 ^a^	
		**3**	0.09 ^a^	0.06 ^a^	0.05 ^a^	0.06 ^a^	0.18 ^abcd^	0.10 ^a^	
		**4**	0.07 ^a^	0.09 ^a^	0.06 ^a^	0.09 ^a^	0.42 ^e^	0.14 ^abc^	
	**Light**	**1**	0.06 ^a^	0.06 ^a^	0.07 ^a^	0.30 ^cde^	0.66 ^g^	0.58 ^fg^	0.02
		**2**	0.08 ^a^	0.09 ^a^	0.08 ^a^	0.18 ^abcd^	0.43 ^ef^	0.31 ^de^	
		**3**	0.09 ^a^	0.04 ^a^	0.06 ^a^	0.16 ^abcd^	0.27 ^bcde^	0.13 ^ab^	
		**4**	0.07 ^a^	0.09 ^a^	0.06 ^a^	0.10 ^a^	0.17 ^abcd^	0.13 ^ab^	
**Mortadella *a*_w_**	**Dark**	**1**	0.954 ^ghij^	0.938 ^bcdefghij^	0.955 ^ghij^	0.953 ^fghij^	0.920 ^abcd^	0.901 ^a^	0.002
		**2**	0.960 ^ij^	0.932 ^bcdefghi^	0.940 ^bcdefghij^	0.954 ^ghij^	0.936 ^bcdefghij^	0.917 ^abc^	
		**3**	0.958 ^ij^	0.923 ^abcde^	0.943 ^bcdefghij^	0.958 ^hij^	0.934 ^bcdefghi^	0.935 ^bcdefghij^	
		**4**	0.951 ^efghij^	0.941 ^bcdefghij^	0.954 ^ghij^	0.958 ^hij^	0.916 ^ab^	0.928 ^abcdefgh^	
	**Light**	**1**	0.954 ^ghij^	0.924 ^abcdef^	0.955 ^ghij^	0.953 ^fghij^	0.946 ^cdefghij^	0.927 ^abcdefg^	0.002
		**2**	0.960 ^ij^	0.923 ^abcde^	0.954 ^ghij^	0.964 ^j^	0.946 ^cdefghij^	0.931 ^bcdefghi^	
		**3**	0.958 ^ij^	0.936 ^bcdefghij^	0.959 ^ij^	0.955 ^ghij^	0.947 ^defghij^	0.932 ^bcdefghi^	
		**4**	0.951 ^efghij^	0.931 ^bcdefghi^	0.959 ^ij^	0.960 ^ij^	0.951 ^efghij^	0.939 ^bcdefghij^	

*Note*: Results are the means of three independent replicates. Means with different letters within each parameter indicate significant differences (*p *< 0.05), according to statistical analysis performed for each combination of packaging system, light/dark condition, and storage time, in line with the significant second‐order interaction (Pack × Light × Time) reported in Table 2.

Abbreviations: MDA, malondialdehyde; pack, packaging; SE, standard error.

The decrease in CO_2_ concentration can be partly explained by its solubilization in the fatty material of the ham (Gill 1988; Nalçabasmaz et al. 2017; Fernandes et al. 2014). On the contrary, under light conditions an increase in CO_2_ levels over time was determined, with Packaging System 1 showing the highest CO_2_ percentage at the end of the storage period (Table 3). In mortadella samples, there was a general slight decrease in CO_2_ over the storage period under both dark and light conditions, with the significantly lower concentration (*p *< 0.05) determined in packaging 1 under dark conditions at the end of storage (Table 3). For mortadella samples, a more pronounced reduction in CO_2_ concentration was observed over time, likely due to the increased solubilization caused by the higher fat content (USDA FoodData Central 2025). For both cooked ham and mortadella samples, packaging 4 demonstrated the best ability to maintain CO_2_ levels compared to alternative solutions, with an average variation of approximately 4% from the beginning to the end of storage (Table 3). In comparison, as reported in Table 3, conventional System 1 showed a 1 % variation, while systems 2 and 3 exhibited variations of 5% and 10%, respectively.

### 3.2 Colorimetric Analysis

**FIGURE 2:**
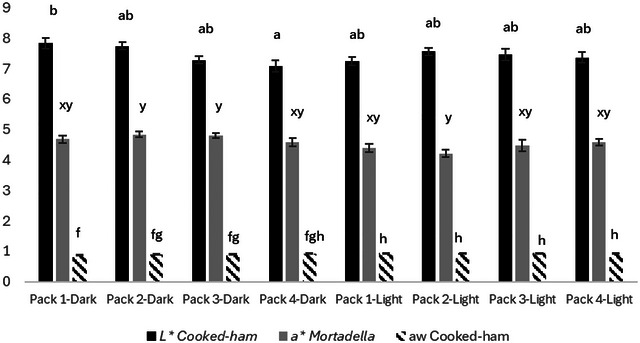
Effect of packaging type and dark/light conditions (in line with the significant first‐order interaction Pack × Light reported in Table 2) on L* and water activity (*a*_w_) of cooked ham samples and a* value of mortadella samples. Data are presented as mean ± standard error. Different superscript letters indicate significant differences (*p *< 0.05) between L* (a‐b), a_w_ (f‐h), and a* (x‐y) values. Pack: packaging.

During the storage of ham, the fading of the red color is mainly due to a combination of light and oxygen, which are capable of causing the photooxidation of the nitrosyl myochromogen (Lloret et al. 2016). When meat is exposed to light in the presence of small amounts of oxygen, it changes to the characteristic gray‐brown color attributed to nitrosyl blood count (Li et al. 2012). In the present study, a significant interaction between the packaging system and the light/dark conditions (*p *< 0.05) was observed with respect to the L* of cooked ham (Table 2). In general, cooked ham stored in Packaging System 1 under dark conditions exhibited the highest L* value (7.83), while those stored in System 4 had the lowest (7.08) (Figure 2).

On the contrary, as reported in Figure 2, no significant differences were observed when samples were stored under light conditions. Regarding a* in cooked ham, a significant interaction between the packaging system and storage period (*p *< 0.05) was observed (Table 2). The high oxygen barrier properties of Packaging System 1, characterized by the material combinations selected for both the tray and lid (OTR value in Table 1), contributed to a better preservation of the red color of cooked ham, with the highest a* value determined on Day 38 (3.99) (Table 4).

**TABLE 4:** Effect of packaging type and storage period (days) on a* and water activity (*a*_w_) value of cooked ham samples.

		Storage period (days)						
	Packaging	0	3	10	22	30	38	SE
Cooked ham a*	**1**	3.13 ^abcd^	2.84 ^ab^	3.91 ^f^	3.95 ^f^	3.83 ^ef^	3.99 ^f^	0.08
	**2**	2.91 ^ab^	2.67 ^a^	3.81 ^ef^	3.64 ^cdef^	3.73 ^def^	3.47 ^bcdef^	0.08
	**3**	2.83 ^ab^	2.95 ^ab^	3.22 ^abcde^	3.65 ^cdef^	3.75 ^def^	3.46 ^bcdef^	0.08
	**4**	3.05 ^abc^	2.74 ^a^	3.44 ^bcdef^	3.74 ^def^	4.00 ^f^	3.70 ^cdef^	0.09
**Cooked ham *a*_w_**	**1**	0.96 ^f^	0.92 ^bcdef^	0.92 ^abcdef^	0.89 ^abc^	0.87 ^a^	0.90 ^abcd^	0.04
	**2**	0.94 ^def^	0.93 ^bcdef^	0.92 ^abcdef^	0.90 ^abcde^	0.88 ^ab^	0.91 ^abcde^	0.01
	**3**	0.94 ^cdef^	0.93 ^bcdef^	0.93 ^bcdef^	0.91 ^abcde^	0.90 ^abcde^	0.91 ^abcdef^	0.01
	**4**	0.95 ^ef^	0.93 ^bcdef^	0.93 ^bcdef^	0.91 ^abcdef^	0.91 ^abcdef^	0.93 ^bcdef^	0.01

*Note*: Means with different superscript letters within each parameter represent significant differences (*p *< 0.05), according to statistical analysis performed for each combination of packaging system and storage time, in line with the significant first‐order interaction (Pack × Time) reported in Table 2.

Abbreviation: SE, standard error.

Interestingly, no significant differences (*p* > 0.05) were observed between the packaging systems from Day 22 until the end of storage (Table 4). In all samples, a significant increase in a* was determined over the storage period (Table 4), as also determined by Park et al. (2016) in salami samples. A similar trend was also observed for b* in cooked ham (Table 5), which was significantly influenced by the storage time (*p *< 0.001) (Table 2).

**TABLE 5:** Effect of storage period (days) on b* value of cooked ham and b* and L* value of mortadella samples.

	Storage period (days)						
	0	3	10	22	30	38	SE
Cooked ham b*	4.49 ^b^	4.08 ^c^	4.93 ^c^	5.15 ^cd^	5.37 ^d^	5.26 ^d^	1.17
Mortadella L*	7.99 ^ab^	7.58 ^a^	7.41 ^a^	8.50 ^b^	8.43 ^b^	8.39 ^b^	0.08
Mortadella b*	6.92 ^bc^	6.28 ^a^	6.55 ^ab^	7.25 ^c^	6.96 ^bc^	6.96 ^bc^	0.06

*Note*: Means with different superscript letters within the same row indicate significant differences (*p *< 0.05), according to statistical analysis performed for storage time in line with the significance reported in Table 2.

Abbreviation: SE, standard error.

The progressive increase in b* reported in Table 5, indicating a shift towards yellow shades, was determined in cooked ham samples and was in agreement with what was reported by Ran et al. (2021). For mortadella samples, both the L* and b* were significantly influenced by the storage time (*p *< 0.001) and the light/dark conditions (*p *< 0.01) (Table 2). Table 5 reports an initial decrease in L* value up to Day 10, followed by a significant (*p *< 0.05) increase until the end of the storage period. This trend aligns with previous studies performed by Ran et al. (2021) and Van Laack et al. (1994) and could be related to the denaturation of myofibrillar and sarcoplasmic proteins. In fact, this structural disruption increased free water content, enhancing the surface reflectance of cooked ham. The b* value in mortadella samples remained relatively stable over the storage period, as reported in Table 5, reaching the highest value at Day 22 of storage before gradually decreasing. In addition, storage performed under light conditions induced a significant reduction (*p *< 0.01) in both L* and b*, indicating the shift in mortadella samples towards darker shades, potentially correlated with lipid oxidation triggered by light exposure (Minelli et al. 2020). In particular, the mean L* values of mortadella samples were 8.3 and 7.8 for storage under dark and light conditions, respectively, while the b* values were 7.0 and 6.7. Regarding a*, it was determined there was a significant interaction between packaging and light/dark conditions in mortadella samples (*p *< 0.05) (Table 2). No specific trend was determined between dark‐ and light‐exposed mortadella (Figure 2). However, as reported in Figure 2, mortadella stored under light conditions in Packaging 2 exhibited the lowest a* value (4.22), which was significantly different (*p *< 0.05) from those stored under dark conditions in systems 1, 2, and 3 (4.68, 4.84, and 4.80, respectively). These results suggest that mortadella samples are generally less susceptible to color degradation related to packaging systems, with all alternatives, except System 2, being comparable to conventional packaging under dark conditions. On the contrary, when storage was performed under light conditions, no significant differences (*p *> 0.05) were observed between the different packaging systems, which presented on average an a* value of about 4.42 (Figure 2).

### 3.3 Water Activity (*a*_w_) and pH

The *a*_w_ of cooked ham was significantly affected by the interactions between packaging and time (*p *< 0.001), as shown in Table 2, with *a*_w_ decreasing over the storage period (Table 4). This decline is a typical physiological result that characterizes processed meat products during their storage (Rubio et al. 2007). In particular, as reported in Table 4, samples packaged in System 1 exhibited the highest variation during the monitoring period, with a decrease in *a*_w_ of approximately 7%. This can be related to the moderate moisture barrier property of Packaging System 1, characterized by a lid with the highest WVTR value (Table 1). On the contrary, cooked ham samples stored in System 4 presented the lowest variation, with an *a*_w_ value decreasing from 0.95 to 0.93 after 38 days of storage (Table 4). However, when comparing samples within the same storage period, no significant differences were observed (*p* > 0.05) (Table 4). A significant interaction between packaging and dark/light conditions (*p *< 0.001) was also observed for *a*_w_ of cooked ham (Table 2), and the results are reported in Figure 2. In general, when comparing samples stored in the same packaging, those kept under light conditions showed significantly higher *a*_w_ (*p *< 0.05) than those stored in the dark (Figure 2). As an exception, Figure 2 reported that cooked ham packaged in System 3 did not show significant differences (*p* > 0.05) between dark (0.92) and light storage conditions (0.93). The lower *a*_w_ identified in samples stored under dark conditions may be related to the relative humidity of the climatic chamber, which was lower than both the product equilibrium relative humidity and the humidity inside the refrigerator used for light storage tests. This was also confirmed by the results reported in Table 6 about the significant interaction between the storage period and the light/dark conditions (*p *< 0.001), again observed in cooked ham samples (Table 2).

**TABLE 6:** Effect of storage period (days) and dark/light conditions on water activity (*a*_w_) of cooked ham and pH and thiobarbituric acid reactive substances (TBARS) of mortadella samples.

		Storage period (days)						
	Dark/ Light	0	3	10	22	30	38	SE
**Cooked ham *a*_w_**	**Dark**	0.948 ^e^	0.923 ^d^	0.914 ^cd^	0.883 ^ab^	0.863 ^a^	0.893 ^bc^	0.004
	**Light**	0.948 ^e^	0.934 ^de^	0.935 ^de^	0.924 ^d^	0.921 ^d^	0.928 ^de^	0.001
**Mortadella pH**	**Dark**	6.28 ^bc^	6.21 ^a^	6.30 ^cd^	6.30 ^cd^	6.28 ^bc^	6.24 ^ab^	0.06
	**Light**	6.28 ^bc^	6.28 ^bc^	6.34 ^d^	6.28 ^bc^	6.29 ^bc^	6.21 ^a^	0.06
**Mortadella TBARS** **mg eq MDA/kg**	**Dark**	0.09 ^ab^	0.12 ^bc^	0.05 ^a^	0.11 ^bc^	0.15 ^cd^	0.20 ^de^	0.07
	**Light**	0.09 ^ab^	0.12 ^bc^	0.11 ^bc^	0.09 ^ab^	0.17 ^de^	0.21 ^d^	0.06

*Note*: Means with different superscript letters within each parameter represent significant differences (*p *< 0.05), according to statistical analysis performed for each combination of light/dark condition and storage time, in line with the significant first‐order interaction (Light × Time) reported in Table 2.

Abbreviation: MDA, malondialdehyde; SE, standard error.

In fact, results in Table 6 demonstrated a more pronounced *a*_w_ reduction under dark conditions, with the significant lower value (*p *< 0.05) identified after 30 days of storage and corresponding to 0.863. This overall decrease in *a*_w_ across the storage period is in agreement with what was reported by Rubio et al. (2006) and Kim et al. (2014) for dry‐cured meat products. In mortadella samples, the *a*_w_ was significantly affected (*p *< 0.001) by the interactions between the packaging system, storage period, and light/dark conditions (Table 2). A general decrease in *a*_w_ was determined from the first day to the end of the storage, which was significant (*p *< 0.05) for samples stored in dark conditions in systems 1 and 2 (Table 3). Furthermore, under dark conditions, mortadella in Packaging System 1 exhibited the highest variation in *a*_w_ during the storage period (Table 3), as also highlighted in cooked ham samples. Conversely, under light conditions, these differences were less pronounced (Table 3). On Day 38, mortadella stored under dark conditions in System 1 presented a significantly lower *a*_w_ (*p *< 0.05) compared to mortadella in systems 2, 3, and 4 under light conditions, as reported in Table 3.

The pH values ranged from 5.34 to 6.46 and are in line with the data reported by Ran et al. (2021). In cooked ham, the pH was significantly influenced (*p *< 0.001) by the interaction between packaging material, storage time, and light/dark conditions (Table 2). In general, a significant decrease in pH was observed during the storage period, with the lowest value (*p *< 0.05) determined in samples stored under light conditions in System 1 at days 30 and 38 (Table 3). The data obtained are in line with the results currently present in the literature, which describe the lowering of pH as a natural consequence of product deterioration (Lloret et al. 2016; Ameer et al. 2022). The decrease in pH, reported in Table 3, was more pronounced under light conditions in systems 1 and 2, with an average percentage variation of approximately 14%, potentially compatible with the development of lactic acid by the LAB (Ameer et al. 2022). Regarding mortadella samples, pH was significantly influenced (*p *< 0.01) by the interaction between light and time (Table 2). In particular, a slight decrease in pH was determined during the storage period, which resulted in being slightly more pronounced when samples were stored under light conditions (Table 6).

### 3.4 Lipid Oxidation Measurement (TBARS assay)

Lipid oxidation is a chemical reaction that causes alterations in taste, color, and aroma as well as the production of toxic compounds. In this study, it was measured with the TBARS assay, which detects advanced and extensive lipid oxidation. In cooked ham, a significant interaction (*p *< 0.001) between packaging, time of storage, and light/dark conditions was determined to influence the lipid oxidation (Table 2). In particular, the extent of lipid oxidation increased during the storage period and became significant (*p *< 0.05) for cooked ham stored under light in systems 1 and 2, due to limited light barrier properties of the materials (Table 3). In particular, in Table 3, it was reported that the highest MDA values were detected in cooked ham in System 1 from 30 days of light storage, with concentrations reaching 0.66 mg eq MDA/kg. However, these values remained lower than 2–2.5 mg eq MDA/kg, established as the accepted limit for the absence of rancidity in meat and meat products (Domínguez et al. 2019). The higher value detected in samples stored under light conditions (Table 3) confirms the strong catalytic effect of light on oxidation reactions (Lloret et al. 2016). Regarding mortadella, a significant interaction (*p* < 0.05) between storage time and light/dark conditions was observed for lipid oxidation (Table 2). In particular, a significant increase (*p* < 0.05) in MDA value was determined during the mortadella storage, with the highest value reached after 38 days of monitoring under light storage, as reported in Table 6.

### 3.5 Microbiological Analyses

Microbiological analysis was performed in order to evaluate the impact of the alternative packaging system on the microbial stability of both cooked ham and mortadella during 30 days of storage (Table 7).

**TABLE 7:** Average levels of microbiological analyses (TMC, Psychrotrophs, LAB, Staphylococci, mould, and yeast) in cooked ham and mortadella packaged in systems 1, 2, 3, and 4 under dark conditions.

Sample	Storage time	Pack type	TMC 30°C (cfu/g)	Psychrotrophs (cfu/g)	LAB (cfu/g)	Staphylococci (cfu/g)	Mould and yeast (cfu/g)
**Cooked ham**	**Day 0**	**1**	<100^a^	<10	<10	<10^a^	<10
		**2**	<100^a^	<10	<10	<10^a^	<10
		**3**	4.50E + 02^c^	<10	<10	4.50E + 02^b^	<10
		**4**	1.00E + 02^b^	<10	<10	<10^a^	<10
	**Day 10**	**1**	3.17E + 03^a^	<10^a^	1.37E + 03^a^	<10^a^	<10
		**2**	1.09E + 05^b^	<10^a^	1.48E + 05^c^	2.00E + 01^b^	<10
		**3**	9.60E + 04^b^	1.35E + 02^c^	7.76E + 04^b^	<10^a^	<10
		**4**	6.50E + 03^a^	7.40E + 01^b^	1.75E + 03^a^	<10^a^	<10
	**Day 22**	**1**	1.20E + 07^bc^	6.60E + 01^a^	1.10E + 07^b^	<10	<10^a^
		**2**	1.30E + 06^b^	8.40E + 01^a^	3.30E + 05^a^	<10	1.00E + 02^b^
		**3**	1.38E + 05^a^	9.85E + 02^b^	2.00E + 05^a^	<10	8.30E + 02^b^
		**4**	2.23E + 08^c^	1.10E + 02^ab^	2.16E + 08^c^	<10	<10^a^
	**Day 30**	**1**	5.30E + 07^ab^	3.30E + 02^a^	4.00E + 07^b^	<10 ^a^	<10^a^
		**2**	1.20E + 07^a^	4.20E + 02^a^	8.60E + 06^a^	<10 ^a^	1.80E + 02^b^
		**3**	8.56E + 07^b^	7.18E + 03^c^	3.92E + 07^ab^	5.80E + 03^b^	5.50E + 03^c^
		**4**	6.40E + 07^ab^	9.30E + 02^b^	6.00E + 07^b^	<10^a^	8.00E + 01^b^
**Mortadella**	**Day 0**	**1**	<100^a^	<10	<10	<10	<10
		**2**	4.30E + 02^b^	<10	<10	<10	<10
		**3**	1.60E + 03^c^	<10	<10	<10	<10
		**4**	8.60E + 02^b^	<10	<10	<10	<10
	**Day 10**	**1**	1.10E + 02^a^	<10	<10	<10^a^	<10
		**2**	3.00E + 02^a^	<10	<10	2.00E + 01^b^	<10
		**3**	1.15E + 03^b^	<10	<10	<10^a^	<10
		**4**	4.80E + 04^c^	<10	<10	<10^a^	<10
	**Day 22**	**1**	3.20E + 02^a^	4.00E + 01^b^	<10^a^	3.15E + 02^b^	<10
		**2**	1.00E + 02^a^	<10^a^	<10^a^	<10^a^	<10
		**3**	2.56E + 03^b^	2.00E + 02^b^	<10^a^	1.41E + 03^b^	<10
		**4**	3.30E + 03^b^	7.70E + 01^b^	5.60E + 02^b^	<10^a^	<10
	**Day 30**	**1**	1.40E + 02^a^	<10^a^	<10^a^	<10^a^	<10
		**2**	3.08E + 03^b^	4.50E + 02^b^	<10^a^	1.42E + 03^b^	<10
		**3**	2.40E + 04^c^	3.10E + 02^b^	<10^a^	2.17E + 04^c^	<10
		**4**	2.70E + 04^c^	1.20E + 02^b^	7.50E + 03^b^	1.74E + 04^c^	<10

*Note*: Data are expressed as cfu/g and are the mean of three independent packaging replicates. Significant differences (*p* < 0.05) between packaging systems for each day of storage are indicated by different letters within the column.

Abbreviations: LAB, lactic acid bacteria; Pack, packaging; TMC, total microbial count.

In general, all cooked ham and mortadella samples showed a progressive increase in TMC levels during the storage period, and significant differences were determined between the packaging systems, with samples stored in systems 3 and 4 presenting significantly higher TMC concentrations (*p *< 0.05). For cooked ham, no significant differences were determined between samples packaged in Conventional System 1 and those stored in the Alternative Packaging 2, which presented the lowest TMC at the end of the monitoring period. Furthermore, Alternative System 2 demonstrated its ability in controlling the microbial contamination also regarding LAB, Staphylococci, mould, and yeast levels. A comparable trend was also observed for mortadella samples, with Packaging System 2 performing better than systems 3 and 4 in significantly controlling bacterial development. This high performance observed in Packaging System 2 could be attributed to the high oxygen and moisture barrier properties of the tray and lid materials, as reported in Table 1. Finally, no growth of Enterobacteriaceae, Clostridia sulphite‐reducing, and *B. cereus* was determined in cooked ham and mortadella samples, with levels always lower than 10 cfu/g during the whole monitoring period, ensuring the microbiological safety of the products.

## 4 Conclusion

In this study, sliced cooked ham and mortadella samples were stored using multilayer monomaterial and partially recycled alternative packaging systems, and their performance was compared with that of a conventional multilayer multimaterial high‐barrier solution, with the aim of assessing their effectiveness in preserving product quality during refrigerated storage. Physicochemical properties and microbiological quality were investigated throughout their 38‐day shelf life during refrigerated storage under both dark and light conditions. The results demonstrated how conventional plastic packaging better preserved the color coordinates of cooked ham samples, while for mortadella samples, packaging systems had less influence on product color, with Packaging System 2 under light conditions providing the most pronounced color degradation. Packaging System 1 exhibited the highest variation in *a*_w_ during storage for both mortadella and cooked ham samples. Meanwhile, samples stored in Packaging System 3 presented the highest *a*_w_ levels, suggesting better moisture retention properties compared to other systems. pH decreased in all samples packaged in all systems throughout the storage period, with light exposure accelerating these changes. Finally, microbiological analysis demonstrated that Alternative System 2 performed similarly to Conventional System 1 in controlling microbial growth during product storage. However, lipid oxidation was more pronounced in systems 1 and 2, particularly under light exposure, confirming their limited light barrier properties. Taken together, Packaging System 2 demonstrated the most promising alternative to conventional packaging for both mortadella and cooked ham, especially in terms of physicochemical parameter preservation and microbial stability.

These findings also underline the trade‐offs that often emerge when shifting towards more sustainable packaging. While mono‐material and recycled systems improve recyclability and reduce environmental impact, they may not always match the performance of conventional solutions in preserving food quality parameters. Therefore, performance limitations must be carefully weighed against sustainability goals when designing food packaging strategies.

The results highlight the importance of deeply exploring the effects of more sustainable packaging materials to maintain product quality, particularly in terms of color stability, moisture retention, oxidation control, and gas exchange dynamics. However, the intrinsic complexity of food products makes it challenging to develop a single packaging solution that meets all preservation needs, suggesting the need for tailored approaches based on product‐specific characteristics. In this context, it will be essential to identify specific key parameters that better mirror the product stability and quality, allowing for the selection of the most suitable packaging materials and technologies. Future studies should also consider the potential interactions between the product and the food contact materials, such as the migration of substances from the packaging to the food matrix and/or changes in aroma and flavor profiles. Understanding these interactions will be critical to ensure the packaging not only protects the product but also preserves its sensory and safety attributes during the entire shelf life.

## Author Contributions

**Giulia Leni**: investigation, software, visualization, data curation, writing – original draft, writing – review and editing, methodology. **Cansu Ekin Bonacina**: software, investigation, visualization, writing – original draft. **Laura Principato**: investigation, validation, formal analysis. **Roberta Galli**: investigation, formal analysis, methodology. **Carlo Negrini**: conceptualization, supervision, investigation, visualization, writing – review and editing. **Giuliano Dallolio**: writing – review and editing, software, supervision, investigation, data curation, visualization. **Andrea Bassani**: visualization. **Giorgia Spigno**: data curation, investigation, supervision, project administration, writing – review and editing, resources, conceptualization.

## Conflicts of Interest

The authors declare no conflicts of interest.
